# A new Fight-or-Flight Pacemaker Mechanism via Ryanodine Receptor abundance and superclustering

**DOI:** 10.1371/journal.pcbi.1014267

**Published:** 2026-05-11

**Authors:** Valeria Ventura Subirachs, Syevda Tagirova, Alexander V. Maltsev, Dongmei Yang, Edward G. Lakatta, Michael D. Stern, Victor A. Maltsev

**Affiliations:** National Institute on Aging, NIH, Biomedical Research Center, Baltimore, Maryland, United States of America; University of Nottingham, UNITED KINGDOM OF GREAT BRITAIN AND NORTHERN IRELAND

## Abstract

The sinoatrial node is the primary cardiac pacemaker. Individual sinoatrial node cells (SANCs) generate spontaneous rhythmic action potentials (APs) that initiate each heartbeat. The mechanism of SANC automaticity and its modulation by autonomic nervous system is based on the coupled function of molecules of both the cell membrane (ion channels, exchangers, and pumps) and the sarcoplasmic reticulum (SR), which generates rhythmic local Ca releases (LCRs). While LCRs are generated by ryanodine receptors (RyRs), the molecular-scale RyR network structure remains unknown. Here we performed single-molecule localization of RyRs via direct Stochastic Optical Reconstruction Microscopy (dSTORM) in rabbit SANCs in basal conditions and 5 minutes after β-adrenergic receptor (βAR) stimulation by isoproterenol. RyRs form clusters of various sizes, with a mean density of 67.7 ± 13.2 RyR/μm^2^. (mean±SEM, n = 6 cells). While the majority of cluster sizes ranged from 3 to 32 RyRs, each cell had a few substantially larger clusters (>76 RyRs), dubbed superclusters. βAR stimulation significantly increased the RyR density to 119.1 ± 22.6 RyR/μm^2^ (8 cells, p ≤ 0.05) and created more superclusters. Our numerical SANC model showed that superclustering substantially decreased the AP cycle length (APCL) by creating Ca release hotspots that initiated larger LCRs under any condition. Increasing RyR density prolonged APCL in the basal state but shortened APCL during βAR stimulation. With no change in RyR network, βAR stimulation of only SR Ca pump and ion currents shortened APCL on average from 414.9 to 284 ms. When realistic higher RyR density and superclustering were added to the model, APCL further shortened to 231.9 ms. Thus, dynamic nanoscale changes in RyR network provide a new powerful pacemaker mechanism.

## Author summary

The heartbeat begins in the sinoatrial node, the heart’s natural pacemaker. During stress or exercise, pacemaker cells accelerate their firing rate via β-adrenergic (“fight-or-flight”) stimulation. Although Ca signals are known to drive this acceleration via electrogenic Na/Ca exchanger, how the underlying ryanodine receptor (RyR) Ca release channels are organized at the nanoscale and how this organization changes during stimulation remain unclear. Here, we used super-resolution microscopy (dSTORM) to visualize individual RyRs in rabbit pacemaker cells. We found that RyRs form heterogeneous clusters that include rare, very large “superclusters.” β-adrenergic stimulation increased overall RyR density, enlarged clusters, and markedly increased the number of superclusters. To understand how these structural changes affect pacemaker function, we developed a computational sinoatrial node cell model in which each RyR was represented explicitly and arranged according to our imaging data. Numerical model simulations revealed that superclusters act as Ca-release hotspots that fire early during diastolic depolarization and recruit neighboring RyR clusters to fire via Ca-induced-Ca-release mechanism. When combined with β-adrenergic enhancement of Ca cycling and membrane currents, the superclustering and RyR abundance synergistically accelerated action potential firing. Together, our results identified dynamic nanoscale RyR network reorganization as a key structural mechanism in fight-or-flight response.

## Introduction

The sinoatrial node (SAN) serves as the heart’s primary pacemaker, with individual sinoatrial cells (SANCs) generating spontaneous, rhythmic electrical signals, action potentials (APs) that initiate each heartbeat [1,2]. The underlying cellular mechanism driving this automaticity includes a complex interplay of multiple signals within a coupled-clock system [3,4], involving molecules on the cell surface membrane (ion channels, exchangers, and pumps), known as a membrane clock, and internal Ca-handling structures, primarily the sarcoplasmic reticulum (SR), known as a Ca clock. The Ca clock has two major components: (i) a Ca pump (SERCA molecules) that pumps Ca from cytoplasm into the SR and (ii) Ca release channels (ryanodine receptors, RyRs) that spontaneously rhythmically release Ca from the SR via local Ca releases (LCRs). The LCRs are complex locally propagating events of different morphologies [5] generated by RyR clusters (also known as Ca release units, CRUs) forming an intricate intracellular network interacting via Ca-induced Ca-release (CICR) mechanism.

The LCRs contribute significantly to the pacemaker rate via activation of electrogenic Na/Ca exchanger (NCX) that timely depolarizes the cell membrane [6,7]. This LCR-NCX-induced membrane depolarization activates near-threshold L-type Ca channels, especially the low-voltage-activated Cav1.3 isoform [8], that generate more depolarization and Ca influx that activates more LCRs, forming a positive feedback loop, known as AP ignition [9]. Thus, the RyR network, especially near the cell surface membrane, where RyRs interact with membrane proteins, is crucially important for the generation, synchronization, and propagation of LCRs that regulate the SANC pacemaker function.

The distribution of RyRs in SANCs has been extensively studied by different imaging techniques and numerical modeling. Our previous studies showed that i) synchronization of stochastic CRUs creates a rhythmic Ca clock, and the transition in LCR characteristics is steeply nonlinear over a narrow range of release current, resembling a phase transition [10]; ii) RyR-NCX-SERCA local crosstalk ensures pacemaker cell function at rest and during the fight-or-flight reflex [11], iii) hierarchical clustering of RyRs is important for CICR propagation [12]; iv) CICR facilitation by heterogeneities in CRU sizes and locations regulates and optimizes cardiac pacemaker cell operation under various physiological conditions [13,14]. While previous studies showed functional importance of fine structure of the RyR network, the RyR cluster locations and sizes were imaged by confocal microscopy [12,15,16] and more recently by structured illumination microscopy [14] which cannot resolve individual RyR channels. On the other hand, previous electron microscopy studies did resolve individual RyRs located in peripheral couplings in SANCs [17], but did not describe the RyR network in sufficient detail to glean further functional insight.

Direct Stochastic Optical Reconstruction Microscopy (dSTORM) is a super-resolution imaging technique capable of localizing individual molecules with approximately 20 nm resolution [18]. Importantly, the spatial arrangement of RyR clusters, including their size, packing density, and proximity to neighboring clusters, is now recognized as a key determinant of Ca release properties, with cluster dispersal in disease linked to altered spark dynamics, dyssynchronous release, and arrhythmogenesis (review [19]). Individual RyRs have been previously detected by dSTORM only in cardiac ventricular cells [20,21] showing that the RyR network is dynamic [22–25] and that RyR clusters expand and coalesce after application of isoproterenol, a β-adrenergic receptor (βAR) agonist simulating fight-or-flight response [24].

To further understand how RyRs contribute to cardiac pacemaker function, two major topics require clarification: 1) RyR network structure at the molecular scale in SANC and 2) the network changes during βAR stimulation and their contribution to AP firing regulation. To address this knowledge gap, we used dSTORM-based single-molecule localization to examine the RyR distribution in rabbit SANCs, in cell periphery close to the cell membrane. We found that RyR channels are not uniformly distributed but have a few substantially larger clusters, dubbed superclusters. βAR stimulation significantly increased the RyR density and created more superclusters. To obtain functional insights, we performed numerical model simulations using our refined computational SANC model featuring single RyR resolution and incorporating our new experimental results. Our simulations showed that RyR abundance and superclustering are new powerful pacemaker mechanisms; and that the strongest βAR stimulation effect is achieved when all pacemaker mechanisms work together, i.e., increases in RyR density and superclustering are matched with respective increases in SR Ca pumping and membrane ion currents, L-type Ca current (I_CaL_), funny current (I_f_) and rapid delayed rectifier K current (I_Kr_).

## Results

### βAR stimulation increases RyR density and cluster size and shortens nearest neighbor distances

**Fig 1:**
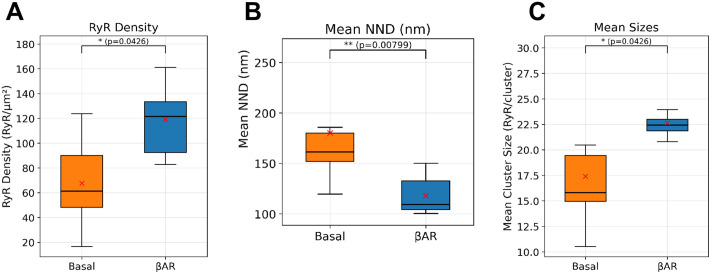
Box-plot comparisons of RyR density, spacing, and cluster size between basal and βAR stimulation. Presented are per-cell metrics from S1 Table derived from DBSCAN-labeled localizations. **A–C.** Box plots show RyR density (RyR/μm^2^), mean nearest neighbor distance (NND, nm) and mean cluster size (RyR/cluster). Box plots display the median (solid horizontal line), interquartile range (box), whiskers extending to 1.5 × the interquartile range. Cross shows mean values. P-values above each panel are from two-sided Mann–Whitney U tests; all comparisons shown are significant (p ≤ 0.05). Together, panels A–C indicate significantly increased density and larger clusters with shorter cluster spacing under βAR stimulation. Cells were treated with 0.3 µM isoproterenol for 5 min at 37 °C; control cells received vehicle only.

We measured and analyzed 14 SANCs, from one rabbit heart, comprising 6 cells in the basal state and 8 cells under βAR stimulation. RyRs form clusters of various sizes, with the mean density of RyRs of 67.7 ± 13.2 RyR/μm² (mean±SEM) in SANCs in the basal state. βAR stimulation significantly increased the RyR density to 119.1 ± 22.6 RyR/μm² and created more superclusters. As summarized in S1 Table, βAR stimulation induced significant changes in the RyR network. RyR density increased by 76.1% (p = 0.04262). Mean cluster size increased by 30.2% (from 17.40 to 22.66 RyR/cluster, p = 0.04262). The 95th percentile of cluster size also increased significantly (p = 0.04262), indicating the emergence of more superclusters. Concurrently, the network became more compact, with the mean nearest neighbor distance between clusters decreasing by 34.5% (from 180.15 nm to 118.02 nm, p = 0.00799). These comparisons are visualized in the box plots in Fig 1. All metrics showed statistically significant differences between the basal and βAR groups (two-sided Mann-Whitney U test, p ≤ 0.05).

### βAR stimulation increases RyR supercluster number and sizes

**Fig 2:**
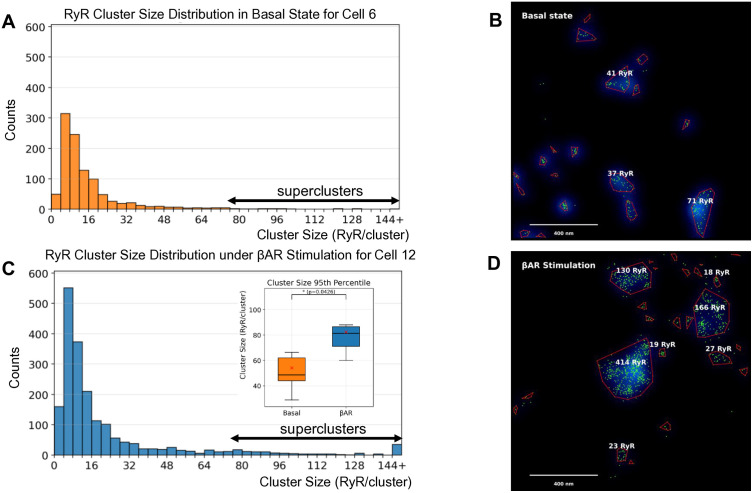
RyR cluster-size distributions and exemplary large clusters under βAR stimulation. Presented are DBSCAN-derived cluster sizes (RyR per cluster) and representative regions from basal and βAR cells. **A, C.** Histograms of cluster size for a basal cell (Cell 6) and a βAR-stimulated cell (Cell 12) show more clusters overall and a right-shifted, heavier tail under βAR, indicating larger aggregates. Double headed arrows show superclusters with sizes >76 RyRs as defined in the text. **B, D.** Examples of the largest clusters in each condition overlaid on a smoothed density map (blue glow); red polygons mark cluster boundaries, numbers indicate RyR per cluster. The basal field contains large clusters (e.g., 71 and 41 RyR), whereas βAR exhibits superclusters reaching 414 RyR. The numbers of clusters and cluster sizes increased during βAR stimulation. Inset in Panel C shows the effect of βAR stimulation on cluster-size within 95th percentile (RyR/cluster) in a box plot. P-value (0.0426) is from two-sided Mann–Whitney U test (see numerical values in S1 Table).

We further examined the increase in cluster size by analyzing the cluster size distributions (Fig 2). In a representative cell in the basal state (Cell 6, Fig 2A), the distribution is highly skewed, with a rapid decay from the smallest cluster sizes. In contrast, the βAR-stimulated cell (Cell 12, Fig 2C) exhibits a right-shifted and heavier tail, indicating a significantly higher population of large aggregates. Panels B and D in Fig 2 show examples of RyR distributions in cell regions in basal state and in the presence of βAR stimulation that clearly visualize the change. While under the basal state, cells contained large clusters (e.g., 71 and 41 RyR), the βAR-stimulated cells featured “superclusters” that were not prevalent in the basal state, reaching sizes up to 414 RyR. This indicates that βAR stimulation promotes the formation of markedly larger RyR aggregates. During βAR stimulation, the 95th percentile of cluster size significantly (p = 0.04262) increased by 52.4%, from 54.09 to 82.43 RyR/cluster (see inset in Fig 2C).

### βAR stimulation changes spatial organization within RyR clusters

RyR clusters were categorized into size groups based on the pooled distribution of cluster sizes across all cells and conditions. We first identified all clusters from 14 cells (6 basal, 8 βAR-stimulated) at the 5-minute timepoint and ranked them by the number of RyRs per cluster. Size categories were defined using percentile cutoffs from this combined distribution:

- Small: ≤ 6 RyRs (≤25th percentile)
- Medium-Small: 7–10 RyRs (25th-50th percentile)
- Medium-Large: 11–21 RyRs (50th-75th percentile)
- Large: 22–76 RyRs (75th-95th percentile)
- Supercluster: > 76 RyRs (>95th percentile), see examples shown by double-headed arrows in Fig 2A and 2C.

This percentile-based approach provides biophysically meaningful categories (as we show below in our SANC model simulations) that capture the natural distribution of cluster sizes while maintaining sufficient sample sizes for statistical analysis.

**Fig 3:**
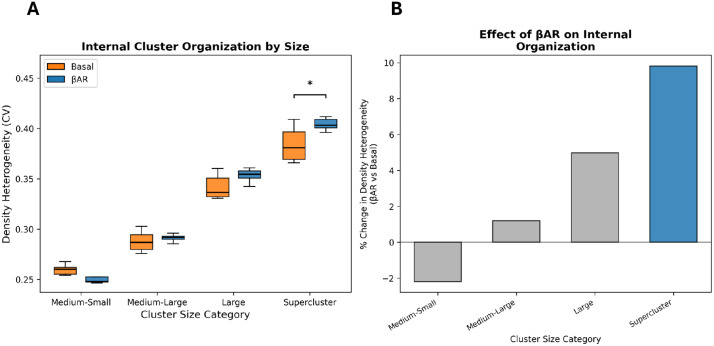
Density heterogeneity analysis reveals size-dependent changes in internal RyR organization following β-adrenergic stimulation. **A.** Box plots showing density heterogeneity (coefficient of variation of local RyR density) for clusters grouped by size category under basal (orange) and βAR-stimulated (blue) conditions. Each data point represents a cell-level mean (n = 6 basal, n = 8 βAR-stimulated cells). Horizontal brackets with asterisks indicate statistically significant differences between basal and βAR groups (Mann-Whitney U test on cell-level means; p ≤ 0.05). Higher CV indicates more uneven internal RyR spacing, consistent with dense cores and sparse boundary regions expected from cluster coalescence. **B.** Percent change in density heterogeneity following βAR stimulation for each cluster size category, showing a size-dependent gradient with the largest effect in superclusters (+9.8%).

If superclusters form through coalescence of adjacent smaller clusters under βAR stimulation; as suggested by the visual evidence in Fig 2, their internal RyR distribution should become less uniform, with the original clusters appearing as dense cores separated by relatively sparse boundary regions at merger sites. To test this prediction, we quantified the coefficient of variation (CV) of local RyR density within each cluster, where higher CV indicates more uneven internal spacing (Fig 3; mathematical details in Methods). Cell-level analysis (n = 6 basal, n = 8 βAR-stimulated cells) revealed that βAR stimulation significantly increased overall density heterogeneity (CV: 0.324 vs. 0.306, p = 0.020, Mann-Whitney U), with the effect being size-dependent (Fig 3A and 3B). Superclusters (>76 RyRs) showed the largest effect, with a 9.8% increase in CV (p = 0.043, Mann-Whitney U), while large clusters (22–76 RyRs) showed a consistent trend (+5.0%, p = 0.108). Smaller cluster categories showed no significant change. This size-dependent pattern is consistent with superclusters forming through coalescence of adjacent clusters under βAR stimulation, as visualized in Fig 2D, creating internal heterogeneity with dense RyR-rich cores separated by relatively sparse regions.

The increasing density heterogeneity with cluster size is consistent with the functional requirement for different cluster sizes to serve distinct roles in cardiac pacemaking, with larger coalesced clusters developing internal sub-domains that may coordinate Ca release across numerous RyRs while avoiding the metastability known for larger fully packed clusters without voids [28].

### Structure-function relationship of RyR network revealed by numerical model simulations

**Fig 4:**
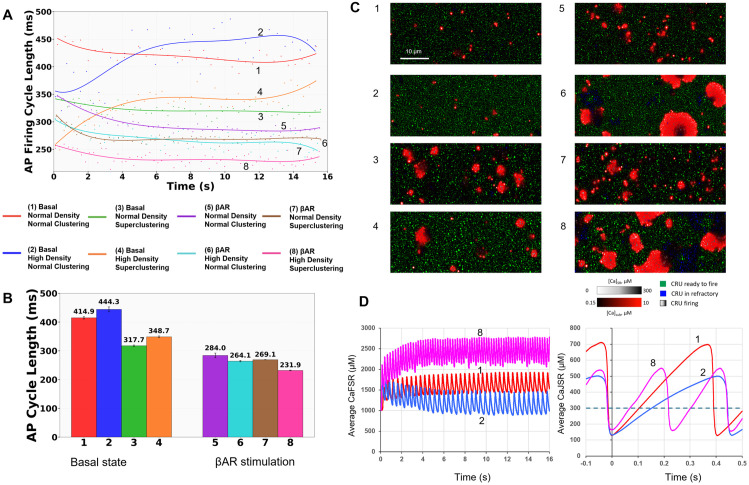
Synergistic effect of RyR density and superclustering on AP firing cycle length during βAR stimulation revealed by numerical model simulations. Shown are simulations for 8 different scenarios (shown by respective numbers at the individual plots and labels) performed for combinations of i) two different RyR densities found at the basal state (in scenarios labeled “normal density” of 67.65 RyR/μm²) and during βAR stimulation (labeled “high density” of 119.07 RyR/μm²); ii) two cluster-size distributions found in the basal state (labeled “Normal clustering” taken from Cell 6, Fig 2A) and during βAR stimulation (labeled “superclustering” taken from Cell 12; Fig 2C); and iii) for basal state or βAR stimulation (labeled “Basal” or “βAR”, respectively). **A.** APCL versus time in all 8 scenarios; solid curves show respective polynomial fits. **B.** Bar plots summarize the steady-state APCL (mean±SD) across all 8 simulation conditions (two densities × two cluster distributions × two coupled-clock states), each run for 5 independent realizations to account for stochastic variability (40 total simulations). See also S1-S8 Videos. **C.** Snapshots of S1-S8 Videos showing submembrane local Ca dynamics and CRUs at −45 mV near ICaL activation threshold for each simulation scenario labeled by numbers at the snapshots. [Ca] was coded by red shades from black (0.15 μM) to pure red (>10 μM). Refractory CRUs are shown in blue shades; CRUs ready to release are in green; and CRUs releasing Ca are in grey shades. Both blue shades and grey shades reflect JSR Ca changes, with a saturation level set at 0.3 mM. Scale bar: 10 µm. **D.** Dynamics of Ca concentration in free SR (CaFSR, left panel) and junctional SR (CaJSR, right panel) in scenarios 1, 2 and 8. In the right panel, the time was shifted to illustrate and compare different rates of junctional SR refilling with Ca from the nadir (t = 0). Horizontal dashed line shows the Ca spark activation threshold (300 μM) in our model.

To further interpret our experimental findings and obtain functional insights into how the RyR network regulates pacemaker function, we performed numerical model simulations. We simulated SANC function for eight scenarios representing all possible combinations of 2 densities (normal and high), 2 RyR clustering (normal clustering and superclustering) and 2 coupled-clock parameter sets, i.e. P_up_ (SR Ca pump), I_CaL_, I_Kr_ and I_f_ for basal state and βAR stimulation. Normal (low) RyR density was set to 67.65 RyR/μm^2^, the average density in the basal state and high density was set to 119.07 RyR/μm^2^, the average density during βAR stimulation (S1 Table). Normal clustering was set as measured in Cell 6 (basal state) and superclustering was set as measured in Cell 12 (βAR stimulation). Results of the simulations are presented in Fig 4. Representative examples of submembrane Ca dynamics together with functional state of each CRU (color coded) are shown in S1-S8 Videos in S1 File for each scenario.

Simulations revealed that increasing RyR density alone prolonged the AP cycle length (APCL) in the basal state firing but shortened APCL during βAR stimulation, a dichotomy explained by differential SR Ca load dynamics (see Discussion). However, introducing superclustering alone shortened APCL under both basal and during βAR-stimulated conditions. The combination of high density and superclustering, representing the full βAR phenotype that we found experimentally, resulted in a synergistic effect that yielded the shortest APCL of all conditions (231.9 ms, magenta color in Fig 4). The critical moment in clock coupling happens close to the threshold of I_CaL_, near the AP ignition onset when LCR activity increases [9]. To get insights into the coupling process, for each scenario we provide snapshots here (Fig 4C) of S1-S8 Videos in S1 File showing submembrane Ca dynamics (coded in red shades) at -45 mV, reflecting LCR activity and their self-organization at that critical moment (Fig 4C). The LCR abundance in the snapshots was inversely linked to APCL: the lowest LCR activity was linked to the longest APCL (slowest AP firing) in basal state with high RyR density and normal clustering (scenario 2), whereas the highest LCR activity was linked to the shortest APCL (fastest AP firing) during βAR stimulation with high RyR density and superclustering (scenario 8). The LCR activity in scenario 8 was self-organized into large and abundant propagating events (S8 Video in S1 File).

Further insights into different LCR activity in different RyR networks were obtained by examining dynamics of Ca concentration in free SR (CaFSR) and junctional SR (CaJSR). Both systolic and diastolic CaFSR levels substantially decreased in scenario 2 with increased RyR density (vs. scenario 1) but substantially increased in scenario 8 with the shortest APCL (Fig 4D, left panel). The CaFSR level decreased in denser RyR network in the basal state, because overall larger junctional SR (accommodating larger RyR numbers) with more connections to free SR shifts source-to-sink balance towards sink. The lower Ca loading of the free SR, in turn, resulted in slower refilling of junctional SR and therefore delayed LCR occurrence during diastolic depolarization as reflected in rare small LCRs in scenario 2 (Fig 4C, right panel).

**Fig 5:**
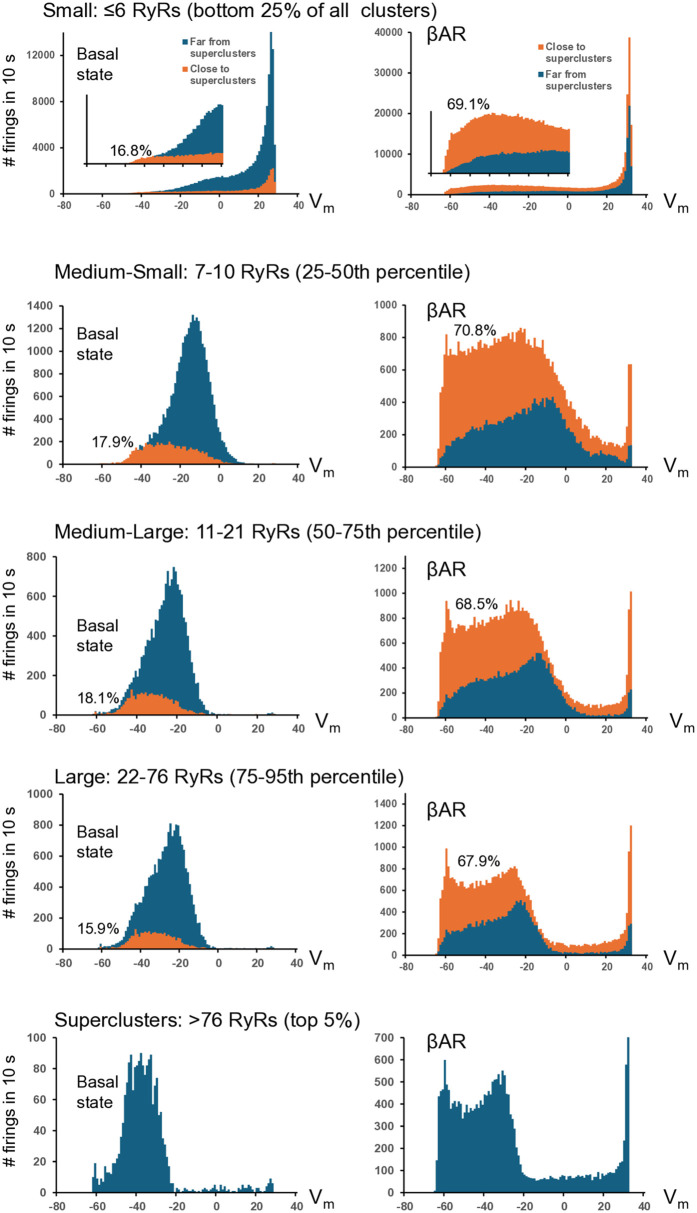
A new mechanism of βAR stimulation via CRU reserve and superclustering revealed by numerical model simulations of SANC function. Shown are results of statistical analysis of numerical simulations of SANC function in basal state (left panels) and during βAR stimulation (right panels) in each CRU size category with respect to membrane potential at which CRUs fire. The analysis was performed separately for CRUs located close to superclusters (with 1 μm neighborhood, orange histograms) and far from the superclusters (dark blue histograms, overlapped). The orange histograms for CRUs near superclusters were shifted to lower membrane potentials (towards diastolic depolarization range) indicating their strong recruitment by superclusters. During βAR stimulation all histograms shifted towards diastolic depolarization range and percentage of CRUs close to superclusters (shown in each histogram) increased resulting in a stronger recruitment of CRUs by the superclusters especially at the very early stage of diastolic depolarization close to maximum diastolic potential near -65 mV.

To learn more about functional importance of different size clusters, we collected statistics on cluster firing averaged across all 5 realizations for each of the 8 scenarios in each size category with respect to membrane potential at which they fire (Fig 5). Histograms of firing events at each potential showed that in the basal state (left panels) a large fraction of smaller clusters began firing at the rapid AP upstroke, i.e., at Vm > -20 mV. Thus, this fraction of smaller clusters stays in reserve and does not contribute to diastolic depolarization. In contrast, a major fraction of larger clusters (Medium-large, Large, and Superclusters) fired during diastolic depolarization (from -65 to -40 mV). Superclusters, being infrequent (>95th percentile), rigorously recruited (via CICR) their neighbors within a 1 μm neighborhood to fire as shown by orange histograms in each size category which were significantly shifted towards lower potentials (almost coinciding with the distribution of superclusters). During βAR stimulation (right panels) firing of all clusters substantially shifted towards diastolic depolarization, indicating that those clusters that were in reserve during basal state now contribute to pacemaker function, representing a new mechanism of βAR stimulation.

How do superclusters participate in this mechanism? First, the network density increases during βAR stimulation, and therefore more clusters fall into the 1 µm neighborhood of the superclusters (67–71% during stimulation vs 16–18% in basal state, Fig 5). Next, Ca releases from clusters become stronger (with higher pumping rate, P_up_), especially in superclusters having more RyRs. Thus, overall recruitment capability of superclusters via CICR substantially increases. This results in a strong recruitment of neighboring RyRs to fire during early diastolic depolarization, creating a pronounced increase in the orange histogram near maximum diastolic potential (MDP, near -65 mV). Other clusters (far from superclusters) fire stronger but at later stages of diastolic depolarization and during AP upstroke.

**Fig 6:**
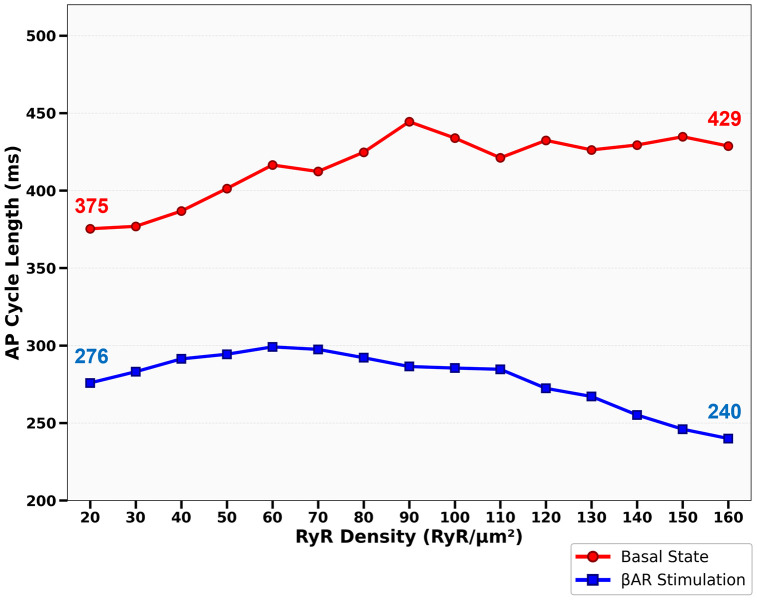
Dependence of AP cycle length on RyR surface density revealed by our numerical model simulations. Shown are steady-state AP cycle lengths (ms) simulated at basal (red) and βAR (blue) settings while varying RyR density from 10 to 160 RyR/μm^2^ with a cluster-size distribution assigned for both βAR stimulation and basal state (Fig 2A).

We further explored the isolated effect of RyR density while fixing the cluster-size distribution of the basal cell (Cell 6, Fig 2A). Under basal conditions, increasing density notably prolonged APCL (from ~375 ms to 429 ms). Conversely, during βAR-stimulation, increasing density shortened APCL (from ~276 ms to 240 ms), accelerating the AP firing rate. This demonstrates that denser CRU networks work synergistically with upregulated Ca cycling, including SR pumping rate and increased membrane currents (βAR state) to shorten diastolic depolarization and increase the AP firing rate (Fig 6).

To evaluate whether our model conclusions are sensitive to the choice of DBSCAN clustering parameter, we repeated all eight simulation scenarios using cluster-size distributions derived with ε multipliers of 3.5 and 4.5 in addition to the default value of 4.0 (S4 Table). S4 Table reports a single representative realization per scenario at each ε value; note that the five-realization means at ε = 4.0 reported in Fig 4B differ slightly due to stochastic variability between runs. Across all three ε values, scenario 8 (βAR stimulation with high density and superclustering) consistently produced the shortest APCL (230.2, 238.0, and 229.1 ms for ε = 4.0, 3.5, and 4.5, respectively; mean 232.5 ± 4.0 ms, CV = 1.7%), while scenario 2 (basal state with high density and normal clustering) consistently produced the longest APCL (446.5, 483.8, and 425.7 ms; mean 452.0 ± 24.0 ms, CV = 5.3%). The rank order of APCL across all eight scenarios was nearly perfectly preserved, with only a minor swap between two closely matched βAR scenarios (scenarios 6 and 7, differing by less than 4% in mean APCL: 266.5 vs 269.5 ms) at ε = 3.5. Across all scenarios, the coefficient of variation of APCL ranged from 1.1% to 5.5%, confirming that our conclusions regarding the synergistic effects of RyR density and superclustering on pacemaker rate are robust to moderate variation in the clustering parameter. Importantly, the key finding, that the full βAR phenotype (high density + superclustering + enhanced Ca cycling and membrane currents) produces the fastest firing rate, was invariant across all ε values.

## Discussion

Using dSTORM-based super-resolution imaging combined with numerical modeling, we established the molecular-scale structure of the RyR network and discovered a novel fight-or-flight pacemaker mechanism via dynamic changes in the RyR network. First, we quantified the network structure in basal and βAR-stimulated SANCs (Figs 1 and 2 and S1 Table). We found that βAR stimulation substantially reorganizes the RyR network. RyR density markedly increases, while the nearest neighbor distances between clusters simultaneously decrease and mean cluster size increases. Importantly, the size increase was not uniform; it was characterized by the formation of “superclusters” (Fig 2), which produced a right-skewed size distribution with an extended upper tail. This reorganization was most evident in the 95th percentile of the cluster size distribution, which increased by 52.4%. Our model simulations showed that the strongest βAR stimulation effect is achieved when increases in SR Ca pumping and membrane ion currents are combined with respective synergistic changes in RyR density and superclustering.

The functional expression of RyRs together with SR Ca pumping defines the capability of the Ca clock to generate stronger Ca releases. So far, the best estimate of about 70 channels per μm^2^ of cell surface has been based on a numerical comparison of immunofluorescence intensity of RyR distribution in serial sections of SANCs and ventricular cells [12]. This was an indirect estimate, whereas the direct measurements of the channel density have not been performed. We not only measured RyR density, but also performed numerical model simulations to examine how, in general, RyR density affects the AP firing rate in SANC in different conditions. One might initially expect that a higher density of channels would result in stronger Ca release and higher AP rates via the coupled-clock mechanism [4]. However, this reasoning does not account for the impact on SR Ca loading. Indeed, model simulations showed (Fig 6, red curve) that higher RyR densities can decrease the AP rate (i.e., increase APCL). Our further examination of SR Ca dynamics (Fig 4D) revealed that an increase in RyR density (and the respective increase in junctional SR size accommodating the additional RyRs) shifts source-to-sink balance towards sink in Ca refilling of junctional SR from free SR. When sink increases, the same SR pumping capability (modest in the basal state) results in lower CaFSR levels. This leads, in turn, to slower CaJSR rise during diastolic depolarization and less LCR activity during diastolic depolarization (Fig 4C, scenario 2) and lower AP rate.

Our simulations also revealed a potentially important pathological consequence: increasing RyR density without proportional increases in SR Ca pumping slows pacemaking by depleting SR Ca stores (Fig 6, red curve). This density pump mismatch could contribute to sinoatrial node dysfunction and sick sinus syndrome [41]. Although aging is associated with changes in multiple Ca² ⁺ handling proteins including RyR2 and SERCA2a [42], our model predicts that the relative balance between RyR density and SR Ca pump capacity, rather than the absolute level of either alone, is a critical determinant of pacemaker rate. If age-related remodeling shifts this balance unfavorably, it could contribute to the well-documented decline in maximum heart rate and chronotropic reserve with aging [42]. This prediction is testable through dSTORM imaging of SANCs from aged animals combined with SERCA expression quantification.

During βAR stimulation, SR pumping capability substantially increases via phospholamban phosphorylation [4] (parameter P_up_ in our model) with increased Ca influx via I_CaL_, naturally matching the increased Ca source to the increased sink (with larger RyR density). The SR Ca loading indeed increases in our model (Fig 4D) as shown in previous experimental studies [26]. This leads, in turn, to faster kinetics of junctional SR refilling with Ca (via diffusion from free SR), resulting in higher LCR activity during diastolic depolarization and pacemaker rate in the model (Fig 4, scenario 8 and Fig 6, blue curve) and previous experimental studies [26,27]. Indeed, a CRU can generate a Ca spark when CaJSR reaches a critical level (300 μM in our model) that allows a phase transition of the RyR release channel system from a metastable state [28,29]. This critical level is achieved the earliest during diastolic depolarization in the presence of βAR stimulation, explaining, in part, the highest LCR activity at the I_CaL_ threshold and the shortest APCL (Fig 4).

We also characterized the distribution of cluster sizes (RyRs per cluster), a parameter that had not been previously established. This parameter is important (along with the channel density) for Ca clock function, because activation and termination of a Ca spark critically depend on the number of release channels in the CRU [28–31]. In the literature, we found only preliminary results of dSTORM-based single RyR imaging in rat SANCs published by the Lederer’s group as abstracts [32,33]. They found that RyRs organized into heterogeneous clusters ranging from 15 to 140 RyRs with short (often < 50 nm) edge-to-edge distances between adjacent clusters, suggesting a possibility of inter-cluster signaling. Our findings are consistent with the results of their study.

The size-dependent increase in density heterogeneity under βAR stimulation (Fig 3) is consistent with coalescence of adjacent RyR clusters into larger superclusters, as visualized in Fig 2 and also reported in ventricular myocytes [24]. Under basal conditions (Fig 2B), clusters maintain relatively discrete boundaries with almost uniform internal RyR distribution. Following βAR stimulation (Fig 2D), superclusters appear likely resulting from merger events, creating dense RyR-rich cores separated by relatively sparse regions. This transition from uniform to heterogeneous organization within superclusters can be important to generate early and stronger synchronized release events (recruiting neighboring smaller CRU to fire, Fig 5) while avoiding the metastability known for larger fully packed clusters (without voids) featuring stronger RyR interactions and generating noisy Ca leak [28]. Indeed, simulations of Ca sparks generated by RyR clusters of different geometries [34] showed that larger and denser clusters exhibit higher spark fidelity, i.e., the probability that a spontaneous RyR opening triggers a Ca spark. Therefore, less-dense larger clusters, such as found here (see also [24]) represent functional groupings of several neighboring clusters close enough that Ca diffusion can cooperatively recruit them into a single large spark while remaining less prone to metastability.

Our numerical simulations (Fig 4) isolated the functional impact of two distinct structural changes, density vs. clustering. The simulations revealed that RyR density and superclustering can be viewed as separate but synergistic regulatory mechanisms. Increasing RyR density alone prolonged APCL in the basal state (Fig 6). However, this high density became advantageous during βAR stimulation, where it worked with upregulated Ca² ⁺ cycling and membrane ion currents to shorten the APCL (Fig 6). Our model explicitly incorporates βAR modulation of I_CaL_ (both Cav1.2 and Cav1.3 components) and I_f_, and the present simulations confirm that rate acceleration requires coordinated enhancement of these membrane currents together with RyR network reorganization (Fig 4). This is consistent with Torre et al. [50], who demonstrated experimentally that Cav1.3 and HCN channels are essential mediators of catecholamine-induced heart rate acceleration in mouse SANCs. Our findings add a structural dimension to that picture: the nanoscale expansion and superclustering of the RyR network amplifies the rate response beyond what membrane channel modulation alone can achieve.

Importantly, the “tail fraction” comprised of superclusters (Fig 2C) acted as a potent accelerator in all conditions. Introducing the supercluster distribution (from Cell 12) shortened the APCL under both basal (from 414.9 ms to 317.7 ms) and βAR-stimulated (from 284 ms to 269.1 ms) conditions by creating Ca² ⁺ release “hotspots” that serve as nucleation sites (S1-S8 Videos in S1 File). This two-tiered organization represents an efficient regulation mechanism, allowing SANCs to maintain a stable basal rate while retaining substantial reserve capacity by engaging higher-density networks and larger superclusters during βAR stimulation.

Our results on RyR clustering-superclustering provide a new dimension to the present perspective on RyRs as dynamic molecules that rearrange their location and function commensurate with demand for Ca release under different conditions. RyR channels self‑organize into clusters whose size, packing, and spacing are highly plastic and adaptive to confer optimal CICR excitability. At the dyads, Junctophilin 2 and Bridging Integrator 1 (BIN1) arrange Cav1.2 and RyR2 into CRU super‑clusters [35,36]. Acute β‑AR stimulation tends to enlarge CRUs and raise spark output [24,37], whereas prolonged β‑AR stimulation or heart failure disperses ryanodine receptor clusters in cardiomyocytes [22,38]. Luminal partners (CASQ2–triadin–junctin), accessories (FKBP), phosphorylation state, and the cytoskeleton fine‑tune this flexible architecture, giving the heart a dynamic range for beat‑to‑beat performance until disease shifts the balance [23,25,39,40].

RyR cluster plasticity is not confined to ventricular myocytes. In atrial myocytes from a sheep model of persistent atrial fibrillation, STED imaging revealed that individual cluster size (~15 RyRs) was preserved, but inter-cluster distances shortened and CRUs accumulated more constituent clusters, producing higher spark frequency with slowed kinetics [48]. The mechanism differs from what we observe in SANCs, where βAR stimulation drives both cluster expansion and de novo supercluster formation. In cerebral artery smooth muscle cells, loss of dystrophin enlarged RyR2 clusters coupled to BK channels, amplifying Ca² ⁺ sparks and impairing vasoconstriction [49]; a case in which larger clusters paradoxically promote relaxation rather than contraction, because the downstream effector is a hyperpolarizing K⁺ channel rather than NCX-driven depolarization. In skeletal muscle, RyR1 forms large, crystalline checkerboard arrays that are mechanically coupled to L-type Ca² ⁺ channels and show far less dynamic rearrangement, consistent with the fundamentally different excitation-contraction coupling mechanism. In the sinoatrial node itself, our earlier SIM study [14] identified two CRU subpopulations whose size heterogeneity and spatial disorder enhanced CICR and AP firing rate in simulations, predicting that cluster expansion and coalescence of the kind we report here could maximize the fight-or-flight response. The present dSTORM data confirm that prediction at molecular resolution.

An important question is how overall RyR density beneath the cell membrane can increase following βAR stimulation. Because our TIRF imaging captures only the sub-membrane population (see Limitations), several mechanisms could account for the observed increase. A similar increase in RyR cluster density after isoproterenol application was reported in ventricular myocytes by Scriven et al. [24], who attributed the increase not to cluster fragmentation but to the introduction of new tetramers into the junctional SR via a dynamic, motor protein-dependent insertion/removal mechanism. In ventricular myocytes, this process may be facilitated by BIN1-organized t-tubule microdomains that rapidly recruit phosphorylated RyRs into dyads [47]. However, SANCs largely lack t-tubules and instead rely on peripheral couplings between the junctional SR and the surface membrane [17]. Whether analogous trafficking mechanisms operate at these peripheral couplings remains unknown. Alternative explanations include phosphorylation-dependent coalescence of pre-existing sub-resolution RyR aggregates into detectable clusters, or redistribution of RyRs from deeper intracellular regions toward the sub-membrane space. Significant de novo RyR2 synthesis within the 5-minute stimulation window seems unlikely. Distinguishing among these mechanisms in SANCs will require complementary approaches such as live-cell tracking of labeled RyRs, quantification of total cellular RyR2 expression before and after stimulation, and ultrastructural analysis of peripheral couplings under βAR stimulation.

Regardless of the underlying mechanism, the timeframe of RyR reorganization carries functional implications. Our experimental protocol involved 5 minutes of isoproterenol exposure, consistent with cluster rearrangement timescales reported in ventricular myocytes [24,37]. This suggests that RyR reorganization contributes to the sustained phase of the fight-or-flight response rather than the immediate acceleration, which is primarily driven by rapid phosphorylation of ion channels and Ca handling proteins. Notably, the direction of RyR reorganization appears to be time-dependent: while acute stimulation (5 minutes) promotes cluster expansion and coalescence [24], Shen et al. [22] showed that prolonged exposure (1 hour) drives progressive cluster dispersion in ventricular myocytes. Our 5-minute timepoint captures the early, constructive phase of this reorganization. Whether partial reorganization may begin within seconds of stimulation remains an open question that would require live-cell super-resolution imaging to resolve.

### Limitations and future studies

As noted above, our dSTORM analysis captures structural snapshots at a single timepoint and cannot resolve the dynamics of RyR reorganization. Our absolute RyR counts depend on several assumptions inherent to single molecule localization microscopy, including incomplete antibody labeling (which would underestimate counts), residual over-counting from imperfect merging of fluorophore blinking events despite our grouping and stability filters (see Methods), and the possibility of multiple localizations per RyR homotetramer due to secondary antibody amplification. These factors affect absolute values of NRyR and derived densities. However, because identical labeling, imaging, and analysis protocols were applied to both basal and βAR-stimulated cells, the relative comparisons between conditions, which form the basis of our main conclusions, remain valid despite systematic uncertainty in absolute channel count. This robustness is further supported by our ε sensitivity analysis (S4 Table), which demonstrated that varying the DBSCAN clustering parameter preserved the rank order of all simulation outcomes.

The findings are based on rabbit SANCs. While rabbit is a common model in cardiac pacemaker research, species differences in ion channel expression could differ substantially. Comparative studies in other species, including human SANCs, are needed to establish the generality of these findings. All cells were obtained from a single rabbit heart; inter-animal variability cannot be assessed from the present dataset and will require future studies with multiple animals.

All localizations in our dataset were acquired in TIRF mode, which selectively excites fluorophores within approximately 100 nm of the coverslip-attached cell surface (see Methods). Because the coverslip-attached surface represents the plasma membrane, all detected RyR clusters are by definition located within this thin sub-membrane layer, with no meaningful axial distance variation within our dataset.

We examined only the RyR network beneath the cell membrane. These sub-membrane RyRs are the population most directly relevant to pacemaker function, as they reside at peripheral couplings where junctional SR opposes the surface membrane and L-type Ca channels (primarily Cav1.3 in SANCs [8]) are co-localized. Accordingly, our computational model places all CRUs directly beneath the cell membrane, with each CRU coupled to LTCCs across a 20 nm dyadic space [51]. In contrast to LTCCs, NCX is uniformly distributed in the cell membrane; thus, RyRs interact with NCX locally, but these interactions are not limited to the dyadic cleft. A recent study by Lang et al. [52] reported the subsarcolemmal cleft in SAN cells to be 15 ± 1 nm, close to the value used in our model. Our model assumes tight functional coupling between each CRU and its local complement of membrane ion channels. However, our imaging visualized only RyRs, and whether superclusters maintain the same spatial relationship with L-type Ca channels as smaller clusters remains unresolved. Co-localization studies using dual-color dSTORM to simultaneously map RyR2 and Cav1.3 would clarify whether superclusters recruit additional L-type Ca channels or extend beyond the footprint of existing membrane channel clusters.

Any computational cell model is a simplification of biological reality. Our model incorporated the experimentally measured distributions of cluster sizes, but it does not capture all possible interactions of RyRs within CRUs and with other molecules in time and space. For example, while our CRU agent-based model of SANC can examine effects of RyR densities and cluster sizes, it does not simulate stochastic gating of individual RyR channels or their cooperative interactions within a cluster (e.g., coupled gating, allosteric activation by neighboring channels, or modulation by accessory proteins such as CASQ2 and FKBP). Instead, each CRU is treated as a unitary release element whose Ca spark amplitude scales with cluster size. Therefore, our speculation about the importance of less organized clusters of larger sizes to avoid metastability will need further numerical validation using a more detailed model of CRUs (such as in [28,34]) that includes interactions of individual RyRs within the cluster. Future studies will address the question of how βAR stimulation affects RyRs in the cell interior and whether these changes are different from (or linked to) RyR distribution/density at the cell periphery. Our data analysis suggests that superclusters are likely formed by smaller cluster merger events. However, how specifically the clusters move, coalesce, expand, and join together in periphery regions remains to be investigated. Finally, further studies will clarify regional heterogeneity of supercluster distribution within cells that might relate to pacemaker site localization within SAN tissue.

### Clinical relevance

Our finding that RyR superclusters are particularly important for achieving the highest possible heart rates during β-adrenergic stimulation is clinically relevant. Dysfunctional RyR gating and altered Ca handling are implicated in sinoatrial node dysfunction (SND) and arrhythmias, including sick sinus syndrome [41]. Cardiac aging is associated with a decline in pacemaker function and increased incidence of SND [42]. Our results suggest that disruptions or alterations in the nanoscale organization of RyR network (such as changes in cluster size distribution or density) could be an underappreciated mechanism contributing to SND. Our results suggest that alterations leading to less synchronized Ca release by a RyR network with fewer large clusters or excessive number of small, disorganized clusters could contribute to bradycardia, and in severe cases, sinus arrest. Investigating how RyR cluster architecture changes with age using super-resolution microscopy could provide new insights into age-related SND.

Impaired β-adrenergic responsiveness is common in aging and heart failure. Our data suggest that structural alterations in the RyR network might contribute to this blunted chronotropic response. Therapeutic strategies aimed at preserving or restoring optimal RyR clustering could potentially improve heart rate regulation. For example, age-associated overexpression of BIN1 emerges alongside dysregulated endosomal recycling and disrupted trafficking of RyRs, whereas BIN1 knockdown restores the nanoscale distribution and clustering plasticity of cardiac RyRs [36].

Our results may also have implications for sports physiology. It was shown that the training-induced bradycardia involves intrinsic sinoatrial node remodeling including downregulation of HCN4 channels [43]. The present study raises the possibility that RyR network remodeling could also contribute the bradycardia. Indeed, our results in Figs 4 and 6 demonstrate that increase in RyR density could lead to lower pacemaker rates at rest.

Understanding the importance of RyR clustering might open new therapeutic avenues. While directly manipulating nanoscale protein organization is challenging, strategies aimed at modulating factors that influence RyR clustering or stabilizing functional cluster sizes could potentially be explored for treating SND or improving heart rate control. Further research correlating RyR nanoscale structure with functional readouts in relevant clinical models (e.g., aging animals, models of heart failure) is needed to fully explore these implications.

## Conclusions

Our study utilized super-resolution dSTORM microscopy to reveal, for the first time, the detailed nanoscale organization of ryanodine receptors beneath the membrane of rabbit SANCs. We discovered significant heterogeneity in RyR density and demonstrated that RyRs form clusters with a right-skewed size distribution, characterized by the prevalence of numerous small clusters alongside a smaller subset of large, functionally significant superclusters. Our computational modeling indicated that this specific architecture, particularly the proportion of large clusters, critically determines the efficiency of local Ca release and thereby modulates SANC automaticity. Larger clusters facilitate faster pacemaking under β-adrenergic stimulation by firing earlier during diastole and recruiting smaller clusters to fire, thereby timely synchronizing Ca release among cluster populations of different sizes. Overall, our new mechanism of β-adrenergic stimulation proposes that a substantial population of smaller RyR clusters do not fire during diastolic depolarization but stay in “reserve” to be recruited during stimulation as an additional diastolic Ca signal source that accelerates diastolic depolarization and AP firing rate. Our findings underscore the crucial role of RyR nanoscale organization, in synergy with SR Ca pumping and cell membrane ion channels (I_CaL_, I_Kr_, and I_f_), as a powerful mechanism regulating cardiac pacemaker function and adaptability.

## Materials and methods

### Ethics statement

The present study conformed to the Guide for the Care and Use of Laboratory Animals,

published by the US National Institutes of Health. The experimental protocols were approved

by the Animal Care and Use Committee of the National Institutes of Health (protocol # 457-LCS-2024). A New Zealand White rabbit (Charles River Laboratories, USA) weighing 2.8-

3.2 Kg was deeply anesthetized with sodium pentobarbital (50–90 mg/kg) injected to the central ear vein. The adequacy of anesthesia was monitored in the rabbit until reflexes to ear and tale pinch and jaw tone were lost.

### Enzymatic isolation of individual SANC

SANCs were isolated from a male rabbit, as previously described [44]. The heart was removed quickly and placed in solution containing (in mM): 130 NaCl, 24 NaHCO_3_, 1.2 NaH_2_PO_4_, 1.0 MgCl_2_, 1.8 CaCl_2_, 4.0 KCl, and 5.6 glucose equilibrated with 95% O_2_/5% CO_2_ (pH 7.4 at 35 °C). The sinoatrial node region was cut into small strips (~1.0 mm wide) perpendicular to the crista terminalis and excised. The final sinoatrial node preparation, which consisted of sinoatrial node strips attached to the small portion of crista terminalis, was washed twice in nominally Ca-free solution containing (in mM) 140 NaCl, 5.4 KCl, 0.5 MgCl2, 0.33 NaH_2_PO_4_, 5 HEPES, and 5.5 glucose (pH = 6.9) and incubated on a shaker at 35 °C for 30 min in the same solution with the addition of elastase type IV (0.6 mg/mL; Sigma, Chemical Co.), collagenase type 2 (0.8 mg/mL; Worthington, NJ, USA), Protease XIV (0.12 mg/mL; Sigma, Chemical Co.), and 0.1% bovine serum albumin (Sigma, Chemical Co.). The sinoatrial preparation was next placed in modified “Kraftbruhe” solution, containing (in mM) 70 potassium glutamate, 30 KCl, 10 KH_2_PO_4_, 1 MgCl_2_, 20 taurine, 10 glucose, 0.3 EGTA, and 10 HEPES (titrated to pH 7.4 with KOH), and kept at 4 °C for 1 h in the Kraftbruhe solution containing 50 mg/mL polyvinylpyrrolidone (Sigma, Chemical Co.). Finally, cells were dispersed from the sinoatrial node preparation by gentle pipetting in the “Kraftbruhe” solution and stored at 4 °C.

### Staining and mounting

Isolated rabbit SANCs were plated on #1 glass, 14-mm glass-bottom MatTek dishes pre-coated with laminin (~40 µg/mL) in phosphate-buffered saline (PBS) with 1% penicillin/streptomycin. Cells were rinsed twice with PBS, fixed in 2–4% paraformaldehyde for 10 min at room temperature, rinsed, quenched in 100 µM glycine for 10 min, rinsed again, permeabilized with 1% Triton X-100 in PBS for 15 min, and rinsed twice with 0.1% Triton/PBS. Samples were then blocked in PBS containing 2% IgG-free BSA, 5% normal goat serum, 0.02% sodium azide, and 0.1% Triton X-100 for 60 min to 4 h. Our primary antibody was RyR2 monoclonal antibody (clone C3-33, Thermo Fisher Scientific cat# MA3–916). The primary antibody was diluted (1:100) in blocking buffer and applied for 60 min at room temperature or overnight at 4 °C, followed by four 5–10 min washes in 0.1% Triton/PBS. Then cross-adsorbed secondary antibody F(ab’)2-Goat anti-mouse IgG (H + L), Alexa Fluor 647 (Thermo Fisher Scientific cat# A-21237) diluted 1:200 was applied to cells and incubated for 60 min at room temperature or overnight at 4 °C in the dark, followed by four 5–10 min washes in 0.1% Triton/PBS in the dark and two final PBS rinses. Then the Alexa Fluor 647-labeled SANCs were submerged in an imaging buffer containing 20% VectaShield (H-1000, Vector Laboratories) diluted in Tris-glycerol (5% v/v TRIS 1 M pH 8 in glycerol, Sigma-Aldrich). For experiments involving acute βAR stimulation, before fixation, SANCs were equilibrated 15–20 min at 37 °C in physiological solution containing 1.8 mM Ca² ⁺ , then treated with 0.3 µM isoproterenol for 5 min at 37 °C; control cells received vehicle only.

### Single-molecule detection

The prepared and mounted SANC samples were imaged using ZEISS Elyra 7 super-resolution microscope, equipped with dSTORM single molecule localization microscopy module (SMLM). Fluorophore emission light was collected via an alpha Plan-Apochromat 100 × objective lens and Immersol 518 F fluorescence-free immersion oil (NE = 1.518 at 23°C). The laser beam was adjusted to TIRF incidence to selectively excite fluorophores on the cell surface within approximately 100 nm of the coverslip. Blinks were collected across a 512 × 512 pixel field of view (51.2 × 51.2 μm at 100 nm/pixel) on the cell surface. All original dSTORM data in the form of Raw SMLM Molecule Tables are available online at Harvard Dataverse https://doi.org/10.7910/DVN/T0REJ3.

Single-molecule detections were performed using ZEN Black 3.0 software throughout each acquisition. The peak mask size was set to 9 pixels, defining the window around each candidate molecule that was isolated for fitting. A peak intensity-to-noise ratio threshold of 6 was applied, so that only peaks at least sixfold above the local background were accepted as genuine detections. Each isolated peak was then fit with the software’s point-spread-function model to obtain subpixel coordinates, and positions were stored as floating-point values (rounded to the nearest nanometer for downstream analysis). Subsequent quality filters and analyses were applied as described in the following sections.

### Image analysis

**Fig 7:**
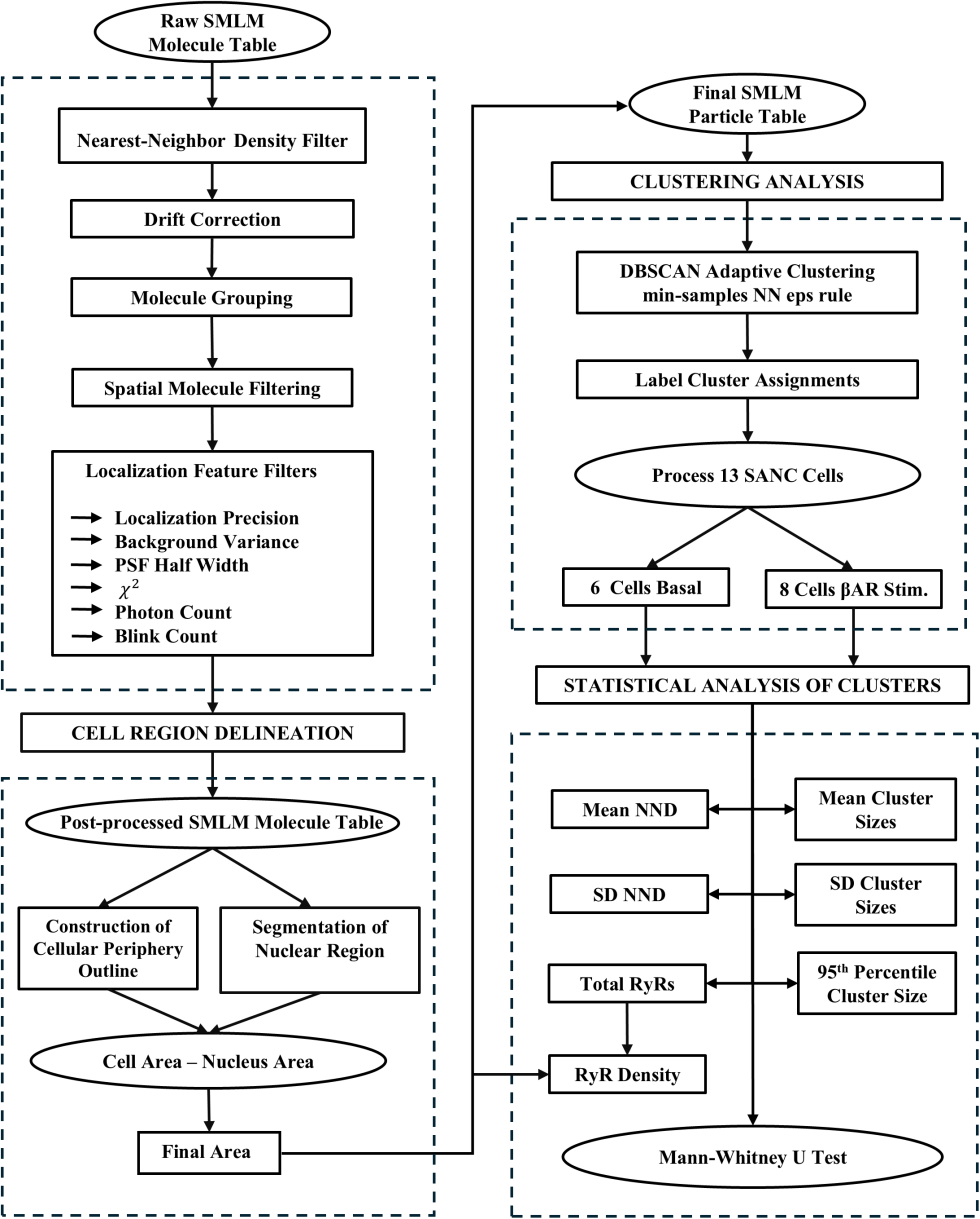
Flowchart of the dSTORM-based RyR cluster analysis algorithm. Presented is our Single-Molecule Localization Microscopy (SMLM) processing pipeline for precise filtering, delineation, clustering, and statistics of RyR localizations in SANCs. Raw detections are drift-corrected, grouped, and spatially filtered, then gated by localization precision (1–30 nm), photon count (1,000–6,000), PSF half-width (6.42–250 nm), χ² (0–2), and background (≤ mean + 1.5·SD), with a ≥ 6 blinks-per-site rule. Cell region is defined by a periphery mask with nuclear-void removal (if applicable) to compute the final area. The post-processed particles are clustered by DBSCAN using an adaptive nearest-neighbor ε; from the labels we derive nearest neighbor distances (NNDs), cluster-size metrics (mean, SD, 95th percentile), and RyR density, and compare groups by two-sided Mann–Whitney U tests.

Our Super-Resolution Single-Molecule Localization Microscopy (SMLM) processing pipeline, depicted in the flowchart in Fig 7 used ZEN Black 3.0 software application supplemented with our Python and MATLAB code (available at GitHub: https://github.com/valventura/dSTORM). The pipeline performs precise filtering, cell region delineation, clustering, and statistical analysis of RyR localizations in SANCs. Our analysis is divided into two major stages: (1) pre-processing and filtering of the *Raw SMLM Molecule Table* generated by the ZEN Black software and (2) clustering and statistical analysis of this final particle table.

### SMLM localization filtering and delineation

**Fig 8:**
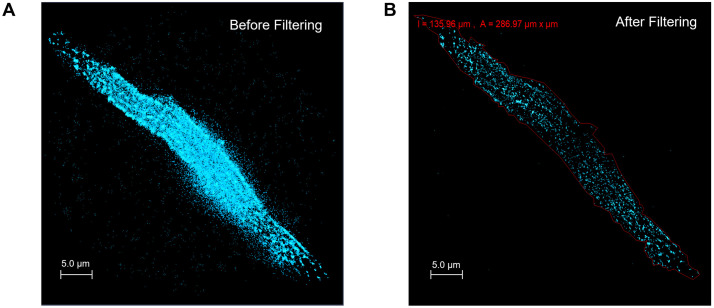
Filtering of RyR dSTORM localizations in a SANC. Shown is a representative cell before and after the filtering pipeline. **A.** Unfiltered localizations (cyan). **B.** Retained localizations (cyan) after applying the periphery mask (red polygon) together with automated windows on localization quality and spatial context. Colors/symbols: cyan dots are RyR events; red polygon is the cell periphery; the red header reports the periphery perimeter (l = 135.96 µm) and area (A = 286.97 µm^2^). Filters applied: precision 1–30 nm, photons 1,000–6,000, PSF half-width 6.42–250 nm, χ² 0–2, background < mean + 1.5 SD; and a blinks-per-site rule (≥6 molecular blinks within 50 nm). Together, panels A–B demonstrate removal of extra-cellular and low-quality detections while preserving the in-cell high-quality RyR localization detections.

**Fig 9:**
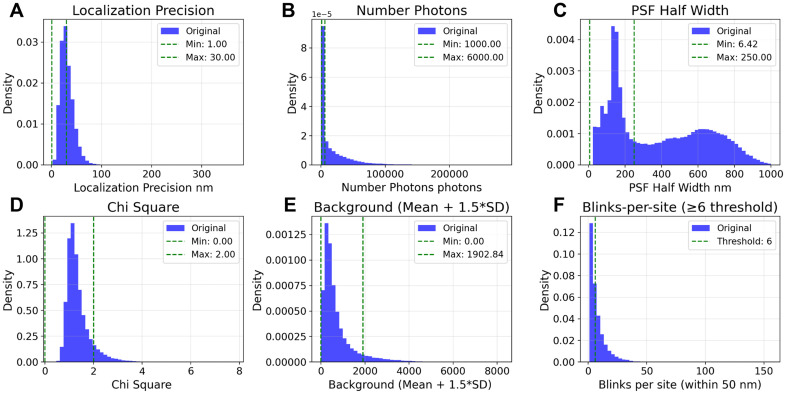
Distributions of dSTORM localization parameters and their filtered values. Density histograms (blue) of the raw localization parameters (calculated for cell 6); green dashed lines mark the acceptance limits used in the filtering pipeline. **A–E.** Filter gates are applied to all detections: localization precision 1–30 nm, number of photons 1,000–6,000, PSF half width 6.42–250 nm, χ² 0–2, and background capped by a dynamic limit of mean + 1.5·SD (0–1902.84 in this dataset). These windows are indicated by the dashed vertical lines in each panel. **F.** Distribution of blinks-per-site computed in a 50 nm neighborhood; the dashed line marks the ≥ 6 blinks threshold used to define stable RyR sites. Together, the figure summarizes the criteria that remove low-precision, low-photon, poorly fit, or high-background events while preserving high-quality in-cell detections; across this cell, 111,307 original localizations were reduced to 13,894 after all gates including the ≥ 6 blinks rule (12.5% retained).

Raw SMLM detections were subjected to a multi-stage filtering process to remove low-quality localizations. An example of filtering within cell area and related histograms are shown in Figs 8 and 9, respectively.

1. Nearest-Neighbor Density Filter

Each localization was evaluated within a circular search area, and if the number of neighboring peaks was below the position threshold, that localization is considered an outlier and removed from the SMLM image and molecule list. For each localization, the number of neighboring molecules within a radius of 100 nm was counted; localizations with fewer than two neighbors were excluded. Single or pairs of isolated RyRs likely represent either non-functional receptors, RyRs not yet incorporated into functional release sites, or artifacts from antibody labeling and imaging.

2. Drift Correction

Sample drift was corrected with the Model‑based method in ZEN Black 3.0. In this approach, the software estimates a linear drift directly from the sample structures. For each segment of the acquisition, a correction vector is computed from the average alignment of all structures. We set Correction Type = Model‑based and enabled segmentation with Segments = Automatic while limiting the max to 4. In automatic mode, the algorithm chose the number of segments but never exceeded the user‑defined maximum. The drift was first corrected within each segment and then residual offsets between segments were corrected.

3. SMLM Grouping

The localization data were grouped in ZEN Black 3.0 using the *SMLM-Grouping* tool to merge repeated detections of the same molecule over consecutive frames. Grouping parameters were chosen Max On Time = 5 frames, Off Gap = 10 frames, and Capture Radius = 1.0 pixels.

The algorithm identifies peaks that fall within the defined capture radius across successive frames and assigns them to a single molecule if the temporal constraints are met.

- *Max On Time* limits the maximum number of consecutive frames in which a fluorophore can appear while still being treated as one molecule. Peaks persisting longer than this are discarded to prevent false merging of overlapping emitters.
- *Off Gap* specifies how many frames the fluorophore may temporarily disappear while still being linked to the same molecule. If this limit is exceeded, the peaks are separated into distinct molecules.
- *Capture Radius* defines the spatial tolerance [set to 1.0 pixel (x, y)] within which peaks are considered part of the same molecule based on the rendered localization precision.

After applying these parameters, the Group function was executed to merge localizations meeting the criteria. This procedure reduced redundant detections from fluorophore blinking, yielding a refined SMLM molecule table with fewer but more accurately positioned molecular localizations.

4. Localization Feature Filters: We applied a series of acceptance windows based on the localization fit parameters, as summarized in S2 Table. These gates included:

- Localization Precision: 1.0–30.0 nm
- Photon Count: 1,000–6,000 photons
- PSF Half Width: 6.42–250.0 nm
- Chi Square ${(\chi }^{\text{2}})$: 0.0–2.0
- Background Variance: A dynamic threshold was applied, retaining localizations with background variance ≤(mean+1.5 × SD) of the cell’s total background distribution.

5. Cell Region Delineation: We analyzed data only within the cell perimeter, i.e., falling inside the periphery polygon (Fig 8). In some images, depending on proximity to the cell surface, nuclei clearly influenced RyR detection, and their respective regions were also excluded from analysis (“Cell Area - Nucleus Area”).
6. Site Stability Filter: To ensure that retained localizations represent stable RyR sites, we combined a spatial neighborhood check with a blink-based stability criterion. First, using a k-d tree, we counted for each localization the number of neighboring localizations within a 50 nm radius. We then computed the number of detected blinks (blinks_per_site) for each site as the total count of temporally linked events assigned to that position. Sites exhibiting fewer than six blinks were excluded from further analysis, an empirically determined threshold indicative of stable fluorophore behavior [45]. A similar filtering algorithm (≥5 neighbors within 60 nm) was used previously in [46]. This two-step filter removed low-confidence, transient detections and retained reproducible RyR localizations (Fig 9).

The set of localizations passing all features, stability, and spatial gates constituted the *Post-processed SMLM Molecule Table*, which was used for all subsequent clustering.

### Clustering analysis

The RyR localizations from the *Post-processed SMLM Molecule Table* were clustered using the Density-Based Spatial Clustering of Applications with Noise (DBSCAN) algorithm to identify RyR clusters. The two key parameters for DBSCAN, min_samples and ε, were determined based on the biophysical characteristics of RyR clusters and the properties of SMLM data.

We defined cluster cores using a min_samples of 3. Given that functional RyR clusters (CRUs) can be small, this value was selected as the minimal robust definition of a dense region (a point and two neighbors). This low threshold provided the necessary sensitivity to detect the smallest biologically relevant RyR aggregates, which may consist of only a few RyR molecules. A higher threshold (e.g., 5 or 10) would risk misclassifying these small but significant nascent clusters as noise, thereby skewing the resulting size and density distributions.

Our calculation of *clustering radius (ε)* in our DBSCAN algorithm is as follows:

1. For a given cell with N localizations, the distance $d_{i}$ to the 3rd nearest neighbor (i.e., corresponding to min_samples = 3) was computed for each point i.
2. From the resulting set of N distances $\{d_{i}$} we identified all positive distances $d_{i}>\text{0}$ and computed their 5^th^ percentile $P_{\text{5}}$.
3. The clustering radius ε is defined as

$$\begin{array}{c} \varepsilon =\text{4}.\text{0}\;\times \;P_{\text{5}}(\left\{d_{i}\;\right|\;d_{i\;}>\text{0}\}) \end{array}$$

**Fig 10:**
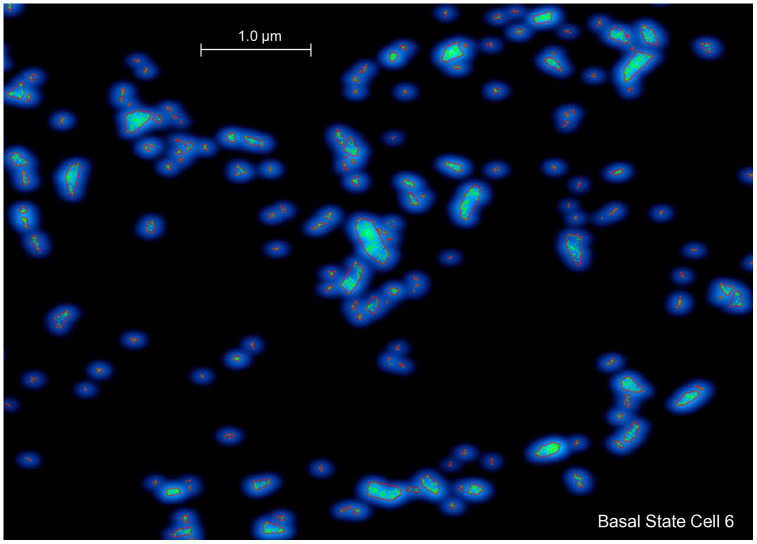
DBSCAN clustering (red outlines) of RyR dSTORM localizations in a basal SANC. A representative example (Cell 6) showing adaptive DBSCAN labels on retained localizations. A smoothed “blue-glow” density map forms the backdrop, lime dots mark RyR events, and red polygons trace convex-hull boundaries of individual clusters. Colors/overlays derive from the plotting routine (blue gradient heatmap + lime points; red hulls). DBSCAN used the min-samples-th nearest-neighbor ε rule (ε = 4.0 × 5th percentile of positive min_samples-th NN distances) with min_samples = 3; points labeled as noise are omitted from the display.

The multiplier 4.0 is dimensionless and was chosen so that ε functions as an interaction envelope: large enough to preserve intra-cluster cohesion across density gradients, yet small enough to maintain inter-cluster separation.. An example result of DBSCAN clustering is shown in Fig 10.

To assess the sensitivity of our results to the clustering parameter ε, we performed additional simulations using cluster-size distributions derived from DBSCAN with ε multipliers of 3.5 and 4.5 (in addition to the default 4.0). These bracketing values were chosen to test whether moderate variation in the clustering radius would alter the functional conclusions of our numerical model.

### Cluster geometry and spacing analysis

Following DBSCAN, we quantified the geometry and spatial relationships of all resulting clusters.

1. Cluster Size and Density: Cluster size was defined as the total number of localizations (RyR) assigned to that cluster label. We computed the mean, standard deviation (SD), and 95th percentile of cluster sizes on a per-cell basis. RyR Density (RyR/μm^2^) was calculated as the total number of clustered localizations divided by the final area.
2. To determine cluster spacing (nearest neighbor distance), we first computed the 2D convex hull for every individual cluster. A global k-d tree was then constructed from all vertices of all hulls in the cell. For each cluster we found the shortest Euclidean distance from any of its hull vertices to a vertex belonging to any other cluster. This shortest inter-hull distance was recorded, and the per-cell Mean and SD of nearest neighbor distance were calculated from this set of inter-cluster distances. Our measured nearest edge-to-edge distances have physiological relevance as RyR clusters interact via CICR mechanism activated when released Ca from one cluster reaches RyRs at the border of a neighboring cluster.
3. Statistical Analysis: Comparisons between basal and βAR-stimulated groups for all derived metrics were performed using a two-sided Mann-Whitney U test.

### Density heterogeneity calculation

To quantify the internal spatial organization of RyRs within individual clusters, we computed the coefficient of variation (CV) of local RyR density across each cluster. For each cluster, local density was estimated at every RyR position using k-nearest neighbor distances: for each RyR, the mean Euclidean distance to its k = 5 nearest neighbors within the same cluster was calculated, and local density was defined as the reciprocal of this mean distance. Clusters containing fewer than 10 RyRs were excluded from this analysis, as reliable estimation of local density variation requires a sufficient number of internal neighbors. The CV of local density across all RyRs within a cluster was then computed as the standard deviation divided by the mean:


$$\begin{array}{c} CV=\frac{\sigma (\rho )}{\mu (\rho )} \end{array}$$

where ρ represents the local density values across all RyRs in the cluster. Higher CV indicates more uneven internal density, as expected when previously separate clusters coalesce to form larger structures with dense cores and sparse boundaries. A CV of zero would indicate perfectly uniform spacing. To respect the nested data structure (multiple clusters within each cell), per-cluster CV values were averaged within each cell to obtain a single cell-level estimate. Statistical comparisons between basal (n = 6 cells) and βAR-stimulated (n = 8 cells) groups were performed using two-sided Mann-Whitney U tests on these cell-level means, both across all clusters and within each size category separately. All cells were obtained from a single rabbit heart.

### Numerical model simulations

Numerical simulations were performed using a refined SANC model featuring a functional network of individual RyRs. We developed this model based on our previous agent-based model in which interacting agents were CRUs [14]. The CRU dynamics in that model were tuned against the Stern et al. Ca spark model [28], which resolves individual RyR channels within a cluster interacting via CICR, and validated against experimental data. Due to voxel size limitations CRU sizes in that model were constructed as multiples of 16 RyRs. In the present study, we overcame this limitation by constructing CRU sizes (and their reciprocal junctional SR sizes and Ca releases) with single-RyR precision, closely reproducing the realistic RyR cluster distributions obtained by our dSTORM imaging. Each CRU functions as an autonomous agent that transitions among three functional states: ready to release, releasing (open), and refractory. A CRU can transition from ready to open only when its local junctional SR Ca concentration exceeds a threshold (300 μM), with the transition probability governed by local Ca^2+^ in the dyadic space. The resulting Ca spark amplitude scales with the number of RyRs in the cluster. Released Ca diffuses to neighboring CRUs, enabling CICR-mediated recruitment. Each CRU is functionally coupled to its local complement of membrane ion channels (I_CaL_, I_NCX_) and the SR Ca pump, and the ensemble of all CRUs drives the global membrane potential via a coupled-clock formulation [4,14].

The modified model code is provided at GitHub: https://github.com/victoramaltsev/RyR-network-SANC-model. The CRUs were randomly rotated and (uniformly) randomly distributed under cell membrane. To minimize effect of randomness on the simulation results, we simulated data for five different random realizations for each simulation scenario of 8 (total 40 simulation runs of 16 s duration) with average data presented in Fig 4B. In each simulation run, APCL data were analyzed for the last 10 s, when the system achieved a steady firing pattern. All model equations, parameters, and their experimental sources are detailed in Appendix A of Maltsev et al. 2024 [14]. βAR stimulation was simulated in the model by modifying four key parameters following: the maximum SR Ca pumping rate P_up_ was increased ×2.0 (from 0.012 to 0.024 mM/ms); the whole-cell maximum I_CaL_ conductance g_CaL_ was increased ×1.75 (from 0.464 to 0.812 nS/pF); the maximum I_Kr_ conductance g_Kr_ was increased ×1.5 (from 0.057 to 0.085 nS/pF); and the midpoint of I_f_ activation (V_1/2_) was shifted +7.8 mV (from −64.0 to −56.2 mV), while I_f_ conductance g_f_ remained unchanged at 0.105 nS/pF (S3 Table). Under basal conditions with realistic CRU distributions, the model produces steady-state AP firing rates of approximately 160 bpm, consistent with experimentally measured rates in isolated rabbit SANCs [14].

## Supporting information

S1 Table Per-cell RyR density, cluster size, and spacing metrics from DBSCAN clustering. (DOCX)

S2 Table Acceptance windows for dSTORM localization quality and site stability. (DOCX)

S3 Table Model parameter values for basal and βAR-stimulated conditions. (DOCX)

S4 Table Sensitivity analysis of AP cycle length to DBSCAN clustering parameter ε. (DOCX)

S1 File S1–S8 Videos 2D Ca dynamics under cell membrane during the last second of each 16 s simulation, with video number corresponding to simulation scenario number from 1 to 8. S1 Video. Basal, Normal Density, Normal Clustering. S2 Video. Basal, High Density, Normal Clustering. S3 Video. Basal, Normal Density, Superclustering. S4 Video. Basal, High Density, Superclustering. S5 Video. βAR, Normal Density, Normal Clustering. S6 Video. βAR, High Density, Normal Clustering. S7 Video. βAR, Normal Density, Superclustering. S8 Video. βAR, High Density, Superclustering. “Basal” designates cell model parameters reflecting pacemaker cell operation in the basal state. “βAR” designates enhanced model parameters P_up_ (SR Ca pump), I_CaL_, I_Kr_ and I_f_ reflecting βAR stimulation. “Normal Density” was set to 67.65 RyR/μm^2^, the average density in the basal state and “High Density” was set to 119.07 RyR/μm^2^, the average density during βAR stimulation (S1 Table). “Normal Clustering” was set to RyR cluster size distribution in Cell 6 (basal state) and “Superclustering” was set to RyR cluster size distribution in Cell 12 (βAR stimulation). [Ca] was coded in videos by red shades from black (0.15 μM) to pure red (>10 μM). CRU functional states were coded by colors as follows: refractory CRUs are in blue shades; CRUs ready to fire are in green; and CRUs releasing Ca are in grey shades. Both blue shades and gray shades reflect JSR Ca changes, with a saturation level set at 300 µM (Ca spark activation threshold in our model). Simulation time and membrane potential are shown at the bottom left corner of each video.
(ZIP)
