# Supplementary material for: A new Fight-or-Flight Pacemaker Mechanism via Ryanodine Receptor abundance and superclustering
Source: PLoS Comput Biol. 2026 May 11;22(5):e1014267. doi: 10.1371/journal.pcbi.1014267 (PMC13178966; doi:10.1371/journal.pcbi.1014267)
Supplement: S3 Table — (DOCX) [file pcbi.1014267.s004.docx]

**S3 Table. Model parameter values for basal and βAR-stimulated conditions.** Key electrophysiological parameters used in the numerical SANC model simulations. Values follow Maltsev et al. 2024 (Cells 13:1885). βAR values represent 100% beta-adrenergic receptor stimulation. (MS Word)

**S3 Table**

| **Parameter** | **Symbol** | **Basal** | **βAR** | **Units** |
| --- | --- | --- | --- | --- |
| Max SR Ca pump rate | P_up_ | 0.012 | 0.024 (×2.0) | mM/ms |
| L-type Ca current conductance | g_CaL_ | 0.464 | 0.812 (×1.75) | nS/pF |
| Delayed rectifier K current | g_Kr_ | 0.057 | 0.085 (×1.5) | nS/pF |
| Funny current conductance | g_f_ | 0.105 | 0.105 | nS/pF |
| Funny current half-activation | V_If,1/2_ | −64.0 | −56.2 (+7.8 mV) | mV |
