# Supplementary material for: A new Fight-or-Flight Pacemaker Mechanism via Ryanodine Receptor abundance and superclustering
Source: PLoS Comput Biol. 2026 May 11;22(5):e1014267. doi: 10.1371/journal.pcbi.1014267 (PMC13178966; doi:10.1371/journal.pcbi.1014267)
Supplement: S4 Table — (DOCX) [file pcbi.1014267.s005.docx]

**S4 Table.** Sensitivity analysis of AP cycle length to DBSCAN clustering parameter ε. Steady-state AP cycle lengths (ms) for all eight simulation scenarios (4 basal, 4 βAR) using cluster-size distributions derived with ε multipliers of 3.5, 4.0 (default), and 4.5. Rank columns indicate the ordering of scenarios from shortest (1) to longest (8) APCL at each ε value. Mean, SD, and CV (%) are computed across the three ε values for each scenario.

| **Scenario** | **Condition** | **ε = 4.0 (ms)** | **ε = 3.5 (ms)** | **ε = 4.5 (ms)** | **Rank 3.5** | **Rank 4.0** | **Rank 4.5** | **APCL mean (ms)** | **SD (ms)** | **CV (%)** |
| --- | --- | --- | --- | --- | --- | --- | --- | --- | --- | --- |
| 1 | **Basal** | 414.39 | 457.80 | 403.56 | 7 | 7 | 7 | 425.25 | 23.44 | 5.51 |
| 2 | **Basal** | 446.54 | 483.79 | 425.73 | 8 | 8 | 8 | 452.02 | 24.02 | 5.31 |
| 3 | **Basal** | 319.19 | 332.28 | 306.75 | 5 | 5 | 5 | 319.40 | 10.42 | 3.26 |
| 4 | **Basal** | 346.72 | 360.17 | 341.92 | 6 | 6 | 6 | 349.60 | 7.73 | 2.21 |
| 5 | **βAR** | 285.97 | 296.23 | 283.01 | 4 | 4 | 4 | 288.40 | 5.66 | 1.96 |
| 6 | **βAR** | 261.77 | 280.74 | 256.93 | 3 | 2 | 2 | 266.48 | 10.27 | 3.86 |
| 7 | **βAR** | 269.29 | 273.23 | 265.97 | 2 | 3 | 3 | 269.50 | 2.97 | 1.10 |
| 8 | **βAR** | 230.21 | 238.03 | 229.14 | 1 | 1 | 1 | 232.46 | 3.96 | 1.70 |
